# Pulmonary rehabilitation to improve physical capacity, dyspnea, and quality of life following pulmonary embolism (the PeRehab study): study protocol for a two-center randomized controlled trial

**DOI:** 10.1186/s13063-020-04940-9

**Published:** 2021-01-06

**Authors:** Stacey Haukeland-Parker, Øyvind Jervan, Hege Hølmo Johannessen, Jostein Gleditsch, Knut Stavem, Kjetil Steine, Martijn A. Spruit, René Holst, Mazdak Tavoly, Frederikus A. Klok, Waleed Ghanima

**Affiliations:** 1 Department of Physical Medicine and Rehabilitation, Østfold Hospital Trust, PB 300, 1714 Grålum, Norway; 2 Oslo Centre for Biostatistics and Epidemiology, Institute of Basic Medicine, University of Oslo, Oslo University Hospital, Oslo, Norway; 3 Department of Internal Medicine, Østfold Hospital Trust (number 3), Grålum, Norway; 4 Department of Health and Welfare, Østfold University College, Fredrikstad, Norway; 5 Department of Radiology, Østfold Hospital Trust, Grålum, Norway; 6 Department of Pulmonary Medicine, Medical Division, Akershus University Hospital, Lørenskog, Norway; 7 Health Services Research Unit, Akershus University Hospital, Lørenskog, Norway; 8 Department of Cardiology, Medical Division, Akershus University Hospital, Lørenskog, Norway; 9 Department of Research and Development, CIRO+, Horn, The Netherlands; 10 Department of Respiratory Medicine, Maastricht University Medical Center (MUMC+), and NUTRIM School of Nutrition and Translational Research in Metabolism, Maastricht, The Netherlands; 11 REVAL Rehabilitation Research Center, BIOMED Biomedical Research Institute, Faculty of Rehabilitation Sciences, Hasselt University, Diepenbeek, Belgium; 12 Department of Medicine, Sahlgrenska University Hospital, Gothenburg, Sweden; 13 Department of Medicine – Thrombosis and Haemostasis, Leiden University Medical Center, Leiden, The Netherlands; 14 Departments of Research, Emergency Medicine and Hematooncology, Østfold Hospital Trust, Grålum, Norway

**Keywords:** Pulmonary embolism, Rrehabilitation, Dyspnea, Exercise capacity, Quality of life, Randomized controlled trial

## Abstract

**Background:**

Recently, a large group of patients with persistent dyspnea, poor physical capacity, and reduced health-related quality of life (HRQoL) following pulmonary embolism (PE) has been identified and clustered under the name “post pulmonary embolism syndrome” (PPS). These patients seem good candidates for pulmonary rehabilitation. The aim of the study is to explore whether a pulmonary rehabilitation program can improve physical capacity, dyspnea, and HRQoL in PPS patients.

**Methods:**

A two-center randomized controlled trial (RCT) is being performed at Østfold Hospital and Akershus University Hospital in Norway. Patients with PPS are 1:1 randomized into an intervention or a control group. The intervention consists of a supervised, outpatient rehabilitation program twice weekly (1 h) for 8 weeks provided by experienced physiotherapists. The intervention involves individually adapted exercises based on existing pulmonary rehabilitation programs (relaxation, interval, and resistance training), and an educational session including topics such as normal anatomy and physiology of the respiratory and circulatory system, information on PE/PPS, breathing strategies, and benefits of exercise/physical activity. Patients randomized to the control group receive usual care without specific instructions to exercise.

Participants in the intervention and control groups will be compared based on assessments conducted at baseline, 12 weeks, and 36 weeks after inclusion using the incremental shuttle walk test (primary outcome) and endurance shuttle walk test (exercise capacity), Sensewear activity monitor (daily physical activity), the modified Medical Research Council scale, the Shortness of Breath Questionnaire (dyspnea), and EQ-5D-5L and the Pulmonary Embolism Quality of Life Questionnaire (HRQoL).

Recruitment of 190 patients is currently ongoing.

**Discussion:**

Results from this study may provide a currently untreated group of PPS patients with an effective treatment resulting in reduced symptoms of dyspnea, improved exercise capacity, and better HRQoL following PE.

**Trial registration:**

Clinical Trials NCT03405480. Registered prospectively on September 2017.

Protocol version 1 (from original protocol September 2017).

The study protocol has been reported in accordance with the Standard Protocol Items: Recommendations for Clinical Interventional Trials (SPIRIT) guidelines (Additional file 1).

## Background

Pulmonary embolism (PE) occurs when an emboli blocks a pulmonary artery resulting in acute symptoms, such as dyspnea and chest pain, which usually subside gradually with the majority of patients regaining normal function within 3–6 months [1]. However, long-term complications following PE can include recurrent venous thromboembolism (VTE), bleeding, and chronic thromboembolic pulmonary hypertension (CTEPH) [2, 3].

Several studies have shown that up to 50% of patients complain of various grades of persistent unexplained dyspnea many years after the diagnosis of PE [4, 5]. Furthermore, patients who reported dyspnea had reduced exercise capacity as measured by the 6-min walk test (6MWT) compared to patients with no dyspnea [6]. Additionally, those suffering from persistent dyspnea had impaired health-related quality of life (HRQoL) compared to both the normative population and PE patients without dyspnea [4]. These findings have recently been confirmed by a prospective study showing that half of PE patients have an exercise limitation at 1 year post-PE which negatively influences walking distance and reduces HRQoL [7]. Some of these patients have persistent pathological findings, such as right ventricular dysfunction, pulmonary hypertension, or residual perfusion defects causing dead space ventilation, which may explain, at least in part, the persistent symptoms. The majority of patients, however, have no detectable cardiopulmonary sequel and merely suffer from deconditioning. Foregoing research has led to the recognition of patients with the so-called post-PE syndrome (PPS), defined as new or progressive dyspnea, exercise intolerance, and/or diminished functional status following PE without an apparent non-PE alternative explanation [8]. Guidelines provide clear recommendations for the management of CTEPH, the most severe presentation of PPS affecting about 4% of the patients following PE [2]. Studies focusing on adequate treatment of other PPS presentations to improve functionality and decrease symptoms are, however, lacking and guidelines make no mention of this large patient group. Because it is likely that physical deconditioning may be responsible for at least a part of the disease burden, it has been hypothesized that patients with PPS may benefit from pulmonary rehabilitation [7, 9].

Pulmonary rehabilitation is a core component in the management of chronic lung disease and is mostly utilized by patients with chronic obstructive pulmonary disease (COPD). Programs typically consist of patient-tailored therapies such as exercise training, education, and behavioral changes, based on a thorough assessment of the patient, with the goal of improving physical and psychological condition and promoting long-term adherence to health-enhancing behaviors [10]. Rehabilitation is a cost-effective intervention and has demonstrated a reduction in respiratory symptoms such as the perception of dyspnea, improved physical function and HRQoL in patients with COPD, and reducing hospital admissions and improving mortality rates [10]. Recently, there has been an increased focus on the benefits of rehabilitation for other types of patients experiencing similar respiratory symptoms and reduced exercise capacity, such as lung cancer, pulmonary hypertension, and cystic fibrosis [10]. Moreover, a study from 2016 investigated the feasibility of a breathlessness rehabilitation program for patients with both respiratory and cardiac disease suggesting that rehabilitation should focus on the symptoms and limitations that patients experience rather than traditional disease-focused rehabilitation [11].

To our knowledge, there are few studies that have addressed the effect and safety of rehabilitation and exercise after PE or DVT. One retrospective study evaluated the safety of rehabilitation after PE, showing that it is safe to start to exercise following PE [12]. One small randomized controlled trial (RCT) objectively measured the effect of exercise and behavioral weight loss after VTE demonstrating that early initiation of exercise was safe and resulted in improvements in physical activity and fitness [13]. Both studies pointed out the need for large prospective RCTs. Furthermore, a recently completed study randomized patients with newly diagnosed PE, regardless of the presence of persistent dyspnea, to a home-based training program or a control group [14]. This study concluded that home-based exercise training and nurse consultations did not improve exercise capacity or symptoms of dyspnea following PE. However, this study included all PE patients, rather than those with PPS only, thus including patients who had recently been diagnosed with PE where a spontaneous improvement in symptoms can be expected from the natural course of the disease. No current studies have provided rehabilitation to patients suffering with PPS. Previous research has indicated that the HRQoL impairment in patients with PPS is driven by reduced physical capacity [4] suggesting a possible receptivity for an intervention such as pulmonary rehabilitation, including exercise training, in order to reduce breathing discomfort and improve HRQoL and exercise capacity.

The aim of this study is to explore the effect of pulmonary rehabilitation on exercise capacity, dyspnea, and HRQoL in patients with PPS.

### Hypothesis

The primary hypothesis is that a structured, outpatient, hospital-based, 8-week pulmonary rehabilitation program will lead to increased exercise capacity, less symptoms of dyspnea, and improvements in HRQoL in patients with PPS as compared to a control group receiving no active intervention.

## Methods and design

### Study design

A two-center RCT is being performed at the outpatient departments of Østfold Hospital Trust (ØHT) and Akershus University Hospital (AHUS) in Norway. Patients with PPS are 1:1 randomized into two arms, an intervention arm and a control arm, using sealed envelopes. The allocation sequence will be computer generated, and to ensure balanced recruitment during the study, this will be performed in blocks of 10. The allocation sequence will not be available to the person enrolling participants and the randomization code will be kept inside sealed opaque envelopes. The generation of the allocation sequence has been performed by the statistician at ØHT. The enrollment process and assignment of interventions will be performed by the PhD candidates.

In addition, a group of patients with no PPS following PE will be examined at baseline to compare patients with and without persistent dyspnea after PE in terms of exercise capacity, daily physical activity, dyspnea, and HRQoL.

The primary study objective is to explore the short-term changes in exercise capacity from baseline to 12 weeks after inclusion between groups as measured by the incremental shuttle walk test (ISWT). The secondary objectives are to explore the long-term effect of the rehabilitation program on exercise capacity 36 weeks after inclusion between groups (ISWT) as well as changes in exercise endurance (ESWT), subjective symptoms of dyspnea, daily physical activity levels, and HRQoL from baseline to 12 and 36 weeks after inclusion.

### Eligibility criteria

Patients diagnosed and treated for PE 6 months to 6 years previously at ØHT or AHUS are identified from ØHT’s Thrombosis registry (TROLL registry—NSD 28435/3/LMR) (ØHT only) or via ICD-10 discharge codes (AHUS). Patients are invited to participate by postal mail.

Inclusion criteria include age 18–75 years, objectively diagnosed symptomatic PE (greater than isolated sub-segmental PE) by CTPA 6 months to 6 years prior to inclusion to the study, persistent dyspnea defined as modified Medical Research Council (mMRC) breathlessness scale grade ≥ 1 that had appeared or worsened after the diagnosis of PE, and the ability to provide written informed consent.

Exclusion criteria include pulmonary diseases (such as COPD GOLD ≥ 2 or restrictive pulmonary diseases, lung cancer, or pleural disease), heart failure, CTEPH, significant valvular heart disease, patients with a condition that would interfere with the ability to comply with the study protocol or to give informed consent (e.g., history of drug abuse, excessive alcohol beverage consumption, cognitive dysfunction, or severe psychiatric disease), active malignancy or recurrent, metastatic or inoperable disease, life expectancy less than 3 months, and pregnancy.

### Blinding

The investigators performing the walking tests at follow-up are blinded to the patients’ group allocation. Due to the nature of the intervention, blinding of the participants and the physiotherapists providing the intervention is not possible. The statistician who will perform the data analysis will be blind to group allocation.

### Intervention

#### Rehabilitation group

Patients in the intervention group are allocated to a basic pulmonary rehabilitation program consisting of a supervised, outpatient exercise program for 1 h twice weekly for 8 weeks. Experienced physiotherapists construct an individually adapted exercise program based on existing pulmonary rehabilitation programs (combining relaxation, interval training at moderate intensity measured with the Borg scale, and resistance training), and an educational session provided by a medical doctor and a physiotherapist. The educational session includes topics, such as normal anatomy and physiology of the respiratory and circulatory system, information on PE and PPS, breathing strategies, and benefits of exercise/physical activity. Training attendance is documented and patients will be given a simple home-based exercise program, consisting of resistance exercises that can be performed without equipment, to be performed once to twice weekly during the intervention period. Implementing supervised, outpatient rehabilitation program will not require alteration to all other usual care pathways (including use of any medication in particular anticoagulation) and these will continue for both trial arms. Minimal actions will be made to improve adherence, for example only one telephone call will be made in the case of poor attendance.

#### Control group

Patients randomized to the control group will receive usual care without specific instructions to exercise (no active intervention). All patients were treated and followed up according to international guidelines [15]. The participants randomized to standard care in the control group will not receive any structured exercise or information as part of the current study, but continue their routine follow-up at the outpatient clinic. However, if they already perform regular physical activity at the time of inclusion, they are encouraged to continue doing so.

### Outcome measures

#### Primary outcome measure

The primary endpoint of the study is improvement in physical capacity as measured by the ISWT. This walking test has been developed to assess exercise capacity and is valid, reliable, and responsive in a number of study populations, including patients with cardiac and respiratory diseases [16]. The patient walks between two shuttles along a 9-m track in a tempo guided by audible sounds which increase in speed every minute for a maximum of 12 min. The test ends when the patient cannot manage to keep the correct speed or has to stop because of symptoms (such as dyspnea or fatigue). Standardized instructions will be provided before the test commences. In order to exclude a learning effect, the ISWT is performed twice at baseline with at least 15 min between tests. Peripheral oxygen saturation is registered and patients will report their subjective experience of dyspnea during exertion using the Borg scale before and immediately after the test [17]. The Borg scale is commonly used for assessing perceived exertion during field walk tests. The minimal clinical important difference (MCID) for the ISWT is 70 m in patients with cardiac disease and 48 m in patients with COPD [18, 19].

#### Secondary outcome measures

##### Endurance shuttle walk test

The endurance shuttle walk test (ESWT) is a derivative of the ISWT. The patient walks between two shuttles along a 9-m track at a predefined speed, usually at 85% of the maximum speed derived from the ISWT. The test ends when the patient cannot continue because of symptoms (such as dyspnea or fatigue) or for a maximum of 20 min (test completion). The outcome of the ESWT is usually reported as time (minutes and seconds), although in some studies the distance completed (meters) has been used. Studies suggest that the ESWT is more sensitive to change after rehabilitation when compared to the 6MWT and ISWT [20, 21]. However, compared to our primary endpoint (ISWT), there is less evidence on using the ESWT and there are no reference values for the PPS population. The MCID for the ESWT has been demonstrated to be 174 to 279 s in COPD after pulmonary rehabilitation [22].

##### Modified MRC dyspnea scale

The mMRC scale is a widely used tool for evaluating the limitation of activities due to dyspnea. This short questionnaire consists of five statements describing the patient’s respiratory disability, ranging from 0 (“not troubled by breathlessness except on strenuous exercise”) to 4 (“too breathless to leave the house or breathless when dressing or undressing”). The MCID for the mMRC is 0.5 points [23].

##### The Shortness of Breath Questionnaire

The Shortness of Breath Questionnaire (SOBQ) is a patient-reported outcome measure which assesses subjective symptoms of dyspnea associated with activities of daily living (ADL). The SOBQ includes 24 items and each is scored on a scale from 0 (“not at all”) to 5 (“maximal/unable to do because of breathlessness”). Total scores range from 0 to 120 with a higher score indicating a higher degree of dyspnea.

##### Sensewear activity monitor

Daily physical activity is measured using a Sensewear activity monitor. The participants will wear the monitor for 1 week before and 1 week after the intervention period to investigate whether the intervention results in a change in daily physical activity or not by measuring the number of steps taken per day and time spent in different activity intensities. Sensewear is a multisensor activity monitor combining a triaxial accelerometer and is shown to be a reliable and valid tool for measuring physical activity in people with respiratory disease [24, 25].

##### EQ-5D-5L

The EQ-5D-5L has been developed by the Euroqol Group as a patient-reported outcome measure to assess generic health status and HRQoL in 5 different dimensions: mobility, self-care, usual activities, pain/discomfort, and anxiety/depression. Each dimension has 5 possible answers ranging from 1 to 5 with a higher score indicating worse possible state, and these scores can be aggregated to a utility score on a 0–1 scale using a tariff of preferences derived from a general population [26]. In addition, the patient subjectively scores their general HRQoL on a visual analogue scale from 0 (“worst imaginable state of health”) to 100 (“best imaginable state of health”).

##### Pulmonary Embolism Quality of Life Questionnaire

The Pulmonary Embolism Quality of Life Questionnaire (PEmb-QoL) is a disease-specific patient-reported outcome measure to assess HRQoL following PE [27]. The PEmb-QoL has 40 items over 6 domains, which assess symptom frequency, the time of day when complaints are at their worst, and the effect of pulmonary-specific symptoms on ADL and work-related problems. Scores for each domain range from 0 to 100 with the average score of all six domains being used to calculate the total score. A lower score indicates better HRQoL. The MCID for the PEmb-QoL is 15 points [28].

##### The Hospital Anxiety and Depression Scale

The Hospital Anxiety and Depression Scale (HADS) is a patient-reported outcome measure assessing symptoms of depression and anxiety. The HADS provides a total score with a 0–42 range with a higher score indicating that the patient is more symptomatic. Scores of ≥ 19 points indicate symptoms corresponding to cases of anxiety and depression, whilst scores between 15 and 18 points suggest possible symptoms of anxiety and depression. It is also possible to calculate a score for anxiety or depression only (range 0–21 points). Scores of ≥ 11 points indicate symptoms that can be compatible with anxiety/depression, and 8–10 points suggest possible symptoms of anxiety/depression. The MCID for the HADS has been suggested to be a reduction of 1.3 to 1.8 points in COPD patients undergoing pulmonary rehabilitation [29].

### Data collection

**Fig. 1:**
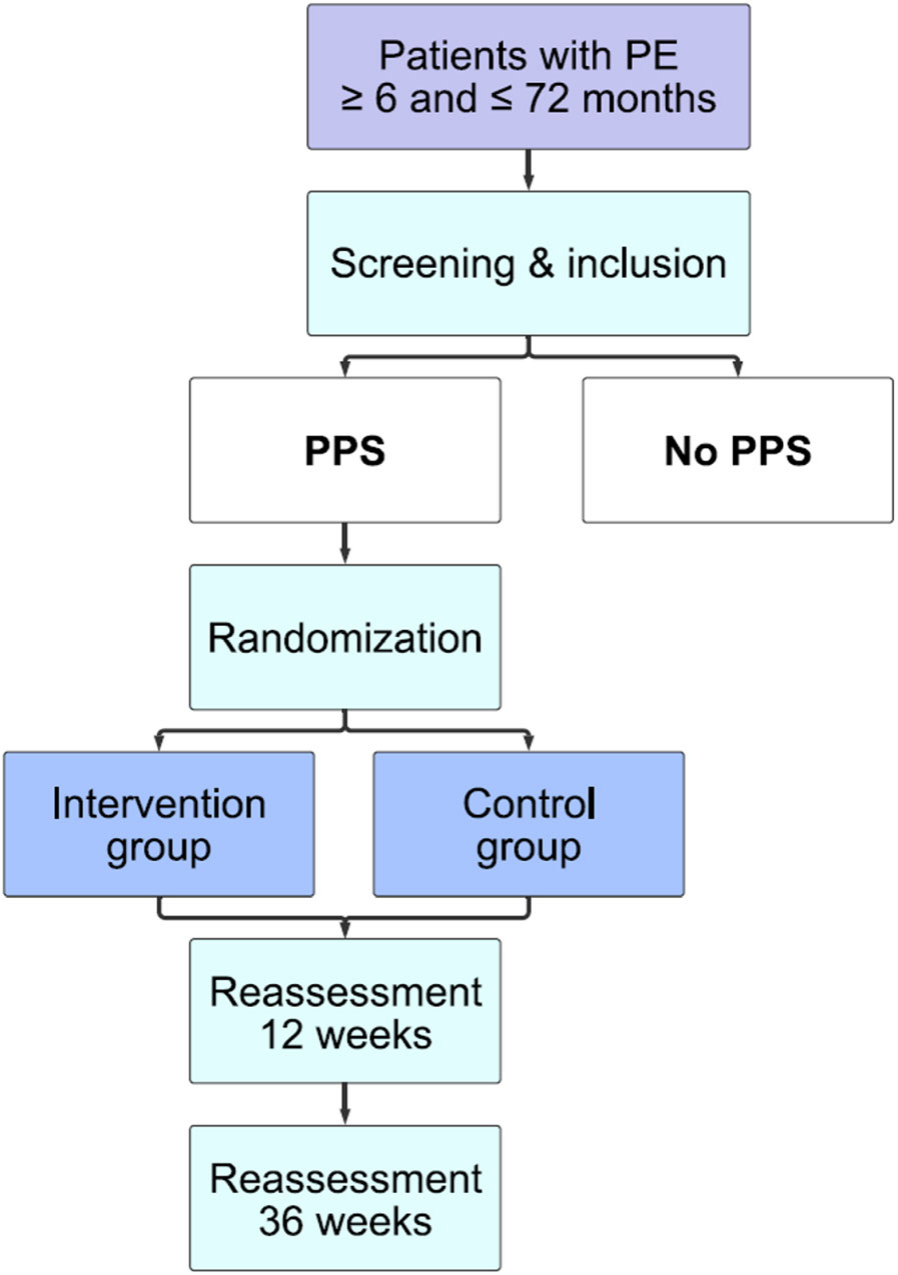
Study design

**Fig. 2:**
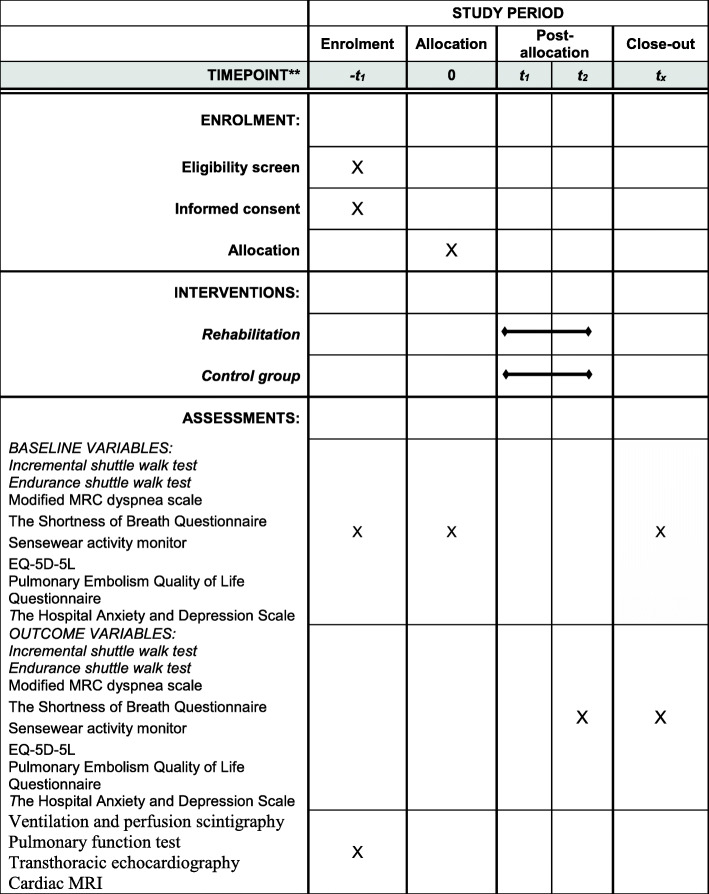
Schedule of enrolment, interventions, and assessments

All outcome measures are completed at baseline and 12 and 36 weeks after inclusion (Figs. 1 and 2). In addition, a complete baseline evaluation is performed on all participants including a full history and medical examination, routine blood tests and biobanking (10 ml EDTA plasma, 10 ml citrated plasma, 10 ml serum, and 10 ml in paxgene), ventilation and perfusion scintigraphy, pulmonary function test (including spirometry, whole body plethysmography, carbon monoxide diffusing capacity of the lung), and transthoracic echocardiography. In addition, cardiac magnetic resonance imaging is performed on 50 participants without PPS and 50 participants with PPS before and after rehabilitation. Finally, patients will be asked to complete questions on self-reported physical activity and exercise habits.

Data collected during the course of the research will be kept strictly confidential and will be stored in the secure research server at ØHT to which only the project investigators have access. Participants will be allocated an individual trial identification number and only de-identified data will be analyzed. The identification key will be stored in a separate file on the secure research server. Only the project investigators and the statistician analyzing the data will have access to the data set. Anonymized data may be shared with other researchers to enable international prospective meta-analyses.

### Data management and analysis

The results will be analyzed according to the intention-to-treat principle. Baseline characteristics will be described by mean and standard deviation, median and interquartile range, or number and proportions as appropriate. The effect of the intervention on the primary outcome (ISWT) will be assessed by comparing the change in exercise capacity after 12 weeks. The primary analysis based on the baseline data and data after 12 weeks will be conducted as a linear regression. The subsequent analysis, which will include data after 36 weeks, will have three measurements per individual to assess the short-term and long-term effect of the intervention and will therefore be analyzed using a linear mixed model. This variant of multiple linear regression allows for addressing such correlations as well as adjusting for possible confounders such as age, body mass index, sex, and treatment center. HRQoL, general activity, and the mMRC breathlessness scale will be compared between the 2 groups at 12 weeks using appropriate statistical tests depending on the normality of data. In addition, the model will also account for missing values. We will apply a post hoc sensitivity analysis to get an indication on potential bias caused by comparing potentially unequal groups with respect to time since PE at the time of inclusion. One way to achieve this is by the use of resampling techniques.

### Sample size calculation

There is currently no data on the physical capacity of PPS patients as measured by ISWT. Therefore, in concurrence with the Danish study that was ongoing when we designed our protocol [30], we have based our sample size calculations on the mean improvement in ISWT previously reported in patients with cardiac and respiratory disease. Rolving et al. assumed that the achieved difference will be around 70 m, i.e., comparable to cardiac patients. Based on 6MWT results from a previous study in patients with PPS [6], patients walked between 413 and 480 m on 6MWT, which is closer to the cardiac population. Therefore, we assume a baseline ISWT for PE patients to be 390 m.

Based on calculations, our clinical experience, and on previous findings, an improvement of 60 m or more on the ISWT will be considered as being of clinical relevance. Given these assumptions, a required sample size to test for that effect size with a type 1 error of 5% and a type 2 error of 20%, 86 patients are needed in each study arm. By adding 10% attrition, the required sample size will be a total of 190 patients. No interim analysis will be performed.

## Discussion

This study is understood to be the first study exploring the effect of structured pulmonary rehabilitation on exercise capacity in patients with PPS. Results from this study may therefore increase the knowledge regarding the management of persistent dyspnea in this patient group as well as providing a currently untreated group of patients with a treatment potentially resulting in reduced chronic symptoms following PE. In comparison to previous studies, patients with chronic, persistent dyspnea from 6 months post-PE are included in the present study in whom no spontaneous improvement may be expected. The ISWT is validated, commonly used in clinical practice, and was the chosen primary outcome for the study by Rolving et al. [30]. Thus, the ISWT was chosen as the primary endpoint in the present study to enable generalization to clinical practice as well as comparison to the findings by Rolving et al.

The definition of PPS is somewhat unclear and several different definitions have been used in previous studies. The study group has chosen to identify PPS patients based on the presence of subjective symptoms of persistent dyspnea, which started or was worsened at the time of PE diagnosis, compared to other studies who have defined PPS as the presence of dyspnea and/or reduced functional capacity and/or reduced HRQoL.

The main inclusion criterion is the presence of a PE event within a period of 6 months to 6 years. Although this timeframe may be considered to be wide, and may potentially result in heterogeneity in the sample population, the study group considered it important that the time since PE should be long enough to prevent the occurrence of spontaneous improvements in dyspnea and physical function following PE as described by Kahn et al. [7]. Our previous research has shown that patients may present with symptoms of dyspnea many years following an acute PE episode; thus, we did not want to deny patients with long standing dyspnea a therapeutic option that may improve their complaint. Further, based on our experience and current research on the effect of pulmonary rehabilitation on patients with chronic dyspnea, the study group chose to include patients with more chronic symptoms as well as those who had suffered with PE relatively recently in order to explore any potential differences in treatment effect between patients with recent PE or more chronic symptoms. In addition, the majority of the participants will be recruited from the ØHT’s Thrombosis registry where few patients will have experienced a PE more than 2 years prior to recruitment; thus, the mean time since PE will be shorter.

Results from this study may have clinical significance by increasing the understanding of the background, assessment, treatment, and prevention of PPS and may change treatment standards in this patient group. The study may also increase the awareness of pulmonary rehabilitation being a feasible treatment for patients with respiratory symptoms similar to COPD and other well-documented respiratory diseases.

### Data monitoring

The study will be monitored by the research department at ØHT. Any adverse effects will be reported. The trial steering committee is made up of the supervisors of the three PhD candidates and a selection of experts within the field of PE. The role of the steering committee is to ensure the quality of the trial and sufficient progress underway. The trial steering committee will meet twice a year to review the progress of the study and address potential challenges and obstacles during the course of the trial. The PhD candidates are responsible for setting up the committee meetings. The group providing day to day support for the progression of the trial is made up of the PhD candidates and their main supervisors, as well as research nurses and research advisors at ØHT who ensure the performance of the trial and practical tasks such as testing patients following intervention (blind to randomization). The PhD candidates are responsible for all aspects of local organization including identifying potential recruits and collecting informed consent. The PhD supervisors and advisors at the research department at ØHT are responsible for supervising the trial and meet regularly. As this trial does not involve any use of pharmaceutical drug or medical device, no formal safety and monitoring board has been established. However, the conduct and progress of the study will be regularly overseen by the leader of the group, professor Waleed Ghanima.

## Trial status

The trial is currently ongoing and recruitment began in January 2018 at ØHT and in August 2019 at AHUS. Recruitment is expected to be complete in late 2020 to early 2021.

Protocol version 1 (original protocol September 2017).

## Supplementary Information

**Additional file 1.** SPIRIT checklist

**Additional file 2.** Ethical approval for the project

## Data Availability

Not applicable, no datasets are included in this study protocol.

## Abbreviations

VTE: Venous thromboembolism
DVT: Deep vein thrombosis
PE: Pulmonary embolism
CTEPH: Chronic thromboembolic pulmonary hypertension
6MWT: Six-minute walking test
HRQoL: Health-related quality of life
PPS: Post pulmonary embolism syndrome
COPD: Chronic obstructive pulmonary disease
RCT: Randomized controlled trial
ØHT: Østfold Hospital Trust
AHUS: Akershus University Hospital
ISWT: Incremental shuttle walk test
mMRC: Modified Medical Research Council breathlessness scale
MCID: Minimal clinical important difference
ESWT: Endurance shuttle walk test
SOBQ: Shortness of Breath Questionnaire
ADL: Activities of daily living
EQ-5D-5L: Five level EQ-5D version
Pemb-QoL: Pulmonary Embolism Quality of Life Questionnaire
HADS: Hospital Anxiety and Depression Scale
