# Supplementary material for: Pulmonary rehabilitation to improve physical capacity, dyspnea, and quality of life following pulmonary embolism (the PeRehab study): study protocol for a two-center randomized controlled trial
Source: Trials. 2021 Jan 6;22:22. doi: 10.1186/s13063-020-04940-9 (PMC7789311; doi:10.1186/s13063-020-04940-9)
Supplement: Supplementary file 2 — Additional file 2. Ethical approval for the project [file 13063_2020_4940_MOESM2_ESM.docx]

**2017/1940 PE-REHAB – Pulmonary rehabilitation following pulmonary embolism**

**Research department:** Østfold Hospital

**Project leader:** Waleed Ghanima

We are writing regarding your application for ethical approval for the project mentioned above. The application has been considered by the Regional committees for medical and health research ethics (REK south-east D) during the meeting on 25.10.2017.

The assessment has been perfomed base don the health research act (hfl.) § 10.

**Project leaders project description**

*Pulmonary embolism (a blood clot in the pulmonary arteries)is a frequent condition with an incidence of approximately 0,5/1000 per year. Recent studies have shown that up to 50% of patients complian of persistent breathlessness and reduced physical capacity following pulmonary embolism despite no objective findings on conventional*

*cardiorespiratory function tests, resulting in these patients being referred to having Post Pulmonary embolism Syndrome (PPS). This project consists of two main parts – an interventions study designed as a randomised controlled trial as well as a pathophysiology study designed as a case-control study. The interventions study will explore the effect of an 8 week*

*rehabilitation program in patients with PPS. If the intervention proves to be effective, this study may provide information about new evidence based treatment for a large patient group who currently have no treatment options.The pathophysiology study will try to bring to light a currently unknown pathophysiological mechanism in PPS with the use of cardiac MRI, ultrasound etc.*

Blood tests which will be taken during the project will be stored in a new research biobank at Østfold Hospital where

Waleed Ghanima is responsible.

**Assessment**

The aim of the project is to explore the effect of an 8 week rehabilitation program in patients with Post

Pulmonary embolism Syndrome (PPS) (randomised controlled study), as well as bring to light information about the pathophysiological mechanisms in PPS (case-control study).

Particiaption involves the collection of information from patient journals and previous diagnostic images, and details from the Trombosis register at Østfold Hospital (information related to pulomary embolism). A clinical assessment will be performed, ECG, walking tests, ventilation-perfusion scintigrafy and low dose CT of the lungs, lung function tests,

Ultrasound of the heart, and use of a activity monitor "Sensewear activity monitor". A small smaple of particiapnts will also perform cardiac MRI and ergospirometry. Blood tests will be taken, and patients will complete questionnaires on quality of life and breathlessness. Included patients are considered to have benifits from participating as pulmonary rehabilitation can result in increased physical capacity and improved quality of life. There are some disadvantages with participation (large number of examinations and use of time), but the risk of complications from the examinations is low.

The commitee considers the advantages to be larger than the disadvantages and the consider it reasonable to perfomed the study as descirbed in the application and protocol.

The commitee have one comment about the project information sheet:

- Under the first point describing which examintations will be performed you have written «Blood tests for biobank». The commitee requests that you provide information about which tests/analysis will be taken as well as the number of tests/analysis that will be taken. You must also include a section about biobanking in accoriding to REK’s template «What happens with the tests that are taken? Tests that are taken from you will be stored in a reaserch biobank [Name of tests that will be stored, Name of biobank, localization of biobank, and who is responsible for the biobank]. The biobank will be destroyed when the project is complete».

The committee sets as condition for approval that the document is revised and forwarded to us.

**Decision**

Based on the heath research act § 9 jf. 33, the commitee approves that the project is performed under the grounds

that terms mentioned above are fulfilled. In addition to the terms mentioned, approval is given under the condition that the project is performed as described in the application and protocol, and the provisions that follow the regulations of the heath research act.

The commitee approves the creation of a sepsific research biobank in line with what is described in the application. The Biobank register will recieve a copy of this letter. If the research biobank ceases, is shut down or taken over by others, an application for permission must be sent to REK according to the health research act § 30.

Permission is valid until 31.12.2027. For the sake of documnetation, the information must be stored until 31.12.2032. The research file must be stored seperatly from a key file. The information must then be destroyed or anonymized within six months from that date.

Data from the research project must be stored responsibly, according to personal data regulations capitle 2, and

The directorate of health’s guide for «Privacy and information scurty in research projects within the healthcare sector».

If considerable changes should be made in the project in terms of the information provided in the application, the project leader must send an application to REK.

The project must send a completion rapport using an own application, within six months of project completion.

The committee’s decision was unanimous.

Appeals

It is possible to appeal against REK’s decision according to the administration act § 28 flg. Appeals should be sent to REK south-east D. Deadline is three weeks from when you receive this letter. If the deision remains the smae from REK south-east D, the appeal is forwarded to the national research ethics for medical and health for final consideration.
